# Compact Dual Circularly-Polarized Quad-Element MIMO/Diversity Antenna for Sub-6 GHz Communication Systems

**DOI:** 10.3390/s22249827

**Published:** 2022-12-14

**Authors:** Sachin Kumar, Sandeep Kumar Palaniswamy, Hyun Chul Choi, Kang Wook Kim

**Affiliations:** 1 Department of Electronics and Communication Engineering, SRM Institute of Science and Technology, Kattankulathur 603203, India; 2 School of Electronic and Electrical Engineering, Kyungpook National University, Daegu 41566, Republic of Korea

**Keywords:** 5G, circularly-polarized, compact, diversity, isolation, MIMO, sub-6 GHz band

## Abstract

In this paper, a compact dual circularly-polarized (CP) planar multiple-input-multiple-output (MIMO) antenna is presented for a sub-6 GHz frequency band. The antenna consists of four identical resonating elements, which are placed in a mirrored-image pattern to obtain polarization diversity. Element 2 is a mirror image of element 1, and elements 3 and 4 are mirror images of elements 1 and 2. Each antenna element comprises an elliptical resonator, a 50-Ω microstrip feed line, and a rectangular stub integrated with the feed to increase the surface current path of the antenna, shifting the resonating frequency to the lower side. Additionally, the rectangular stub is lengthened towards the right side (along the +*x*-axis direction in the antenna element 1), which balances the magnitude and 90° phase variance among the horizontal (*E_x_*) and vertical (*E_y_*) fields. The proposed MIMO antenna supports both types of circular polarization, where radiators 1 and 3 radiate right-hand CP (RHCP) rays and radiators 2 and 4 radiate left-hand CP (LHCP) rays. Developing a compact-size MIMO antenna is a challenging task, especially when the antenna elements share the same ground plane and are placed less than half a wavelength apart. The mutual coupling in the proposed antenna is reduced by increasing the spacing between the elements without the use of any extra decoupling structure. Optimal spacing is maintained to achieve compact geometry with less inter-element correlation. The radiators are closely placed with an edge-to-edge spacing of 0.08*λ*_0_, where *λ*_0_ is the free space wavelength at 3.6 GHz. A peak gain of 5 dBi, efficiency of 90%, an envelope correlation coefficient (ECC) of less than 0.1, and isolation of more than 18 dB are obtained between different ports of the prototype antenna. The overall size of the antenna element is 17 mm × 17 mm × 1.6 mm, and the MIMO antenna is 40 mm × 40 mm × 1.6 mm.

## 1. Introduction

Multiple antenna elements employed at a transceiver help to improve the transmission quality, data rate, and capacity of the wireless system. More radiating elements at a receiver can enhance the link reliability of the receiving system [1]. However, the installation of multiple radiating elements in a small space is a challenging task due to coupling between the antenna elements [2]. In the last few years, a variety of methods have been reported by the researchers to resolve inter-element coupling, utilizing the decoupling element [3], the shielding wall [4], the electromagnetic band-gap (EBG) structure [5], the meta-surface [6], etc. The decoupling structures act to restrict the flow of surface current from an excited port to the other ports. On the other hand, the decoupling element increases the complexity in the design and profile of the multiple-input-multiple-output (MIMO) antenna.

One approach to alleviate the mutual coupling problem is the use of polarization diversity. In [7], mutual coupling between the antenna elements was decreased by arranging the resonators in the vertical and horizontal planes, thus eliminating the need for extra decoupling element(s) to achieve inter-element isolation. A reconfigurable shared aperture MIMO antenna with radiators located in the parallel and perpendicular orientation was presented in [8]. A four-element MIMO antenna with resonators wrapped around a cuboidal polystyrene block was reported in [9]. However, the antenna designs reported in [7,8,9] were complex, non-planar, and relatively bulkier in size. Additionally, linearly-polarized (LP) elements were used to achieve polarization diversity in the aforementioned MIMO antennas. Alternatively, in radio wave propagation, circularly polarized (CP) elements may offer multiple advantages in comparison to the LP elements. CP antennas are less susceptible to multipath fading effects and polarization mismatch amongst the transmitter and receiver [10,11]. Moreover, any change in the alignment of the CP antenna does not affect the signals collected at the terminals. These advantages increase the employability of CP antennas in modern wireless communication systems [12,13].

Recently, a few CP MIMO antennas were presented for satellite navigation, C-band, and WLAN applications [14,15,16,17,18,19,20]. In [14], a two-port antenna was presented for the WLAN band, where the upper CP radiator consisted of a square patch with chamfered corners and 45° slant slot, and the lower LP radiator consisted of a modified inter-digital type structure. In [15], a three-element MIMO antenna was reported for WLAN applications, where the design consisted of a CP square patch and two LP printed dipole antennas. A coplanar waveguide (CPW)-fed square slotted dual CP antenna was presented in [16], which consisted of an inverted-L ground strip and two T-shaped feed lines. A dual CP antenna consisted of a modified ground plane and a radiating patch loaded with L-shaped stubs was presented [17]. A two-port CP MIMO antenna with microstrip line-fed rectangular radiators was proposed in [18], where a mirrored F-shaped defect and three stubs were introduced in the ground plane for high isolation. A CP antenna consisting of two monopole radiators configured as a mirrored image of each other was presented in [19]. A quad-port dual CP antenna was designed in [20], where microstrip line-fed modified G-shaped monopole elements were used to obtain a wide axial ratio bandwidth (ARBW). In [21], a quad-port MIMO/diversity antenna with dual CP characteristics was proposed for the sub-6 GHz band, where two rectangular arms were integrated with the ground plane of the radiating element to realize circular polarization. However, the MIMO/diversity antennas presented in [14,15] could generate LP and right-hand CP (RHCP) waves only. The MIMO antennas presented in [16,17,18,19,20,21] showed dual circular polarizations, but they had defected ground planes and large antenna sizes. Additionally, the ground planes of the antenna elements of the aforementioned MIMO antenna designs were not connected and hence might not be practical in actual applications [22]. Moreover, in the above-mentioned planar antenna designs, the electric currents were distributed both on the resonating elements and on the ground plane(s) so that the radiations from the ground planes were unavoidable. Defect structures on the ground plane can substantially affect the performance of the MIMO/diversity antenna, and a four-port dual CP MIMO antenna with a whole (no-defect) ground plane is hardly reported in the open literature.

In this paper, a small-size dual CP quad-element planar MIMO antenna is designed for a sub-6 GHz band of 3.4–3.8 GHz [23]. Each element of the MIMO antenna consists of an elliptical-shaped patch, which is fed by a 50-Ω microstrip line. To achieve polarization diversity in the proposed antenna, the two antenna elements (1 and 2) are placed in parallel orientation with an edge-to-edge spacing of 0.08*λ*_0_, where element 2 is a mirror image of element 1, and the other two antenna elements (3 and 4) are placed parallel and are configured as the mirror images of antenna elements 1 and 2. Resonators 1 and 3 radiate RHCP waves, and resonators 2 and 4 radiate left-hand CP (LHCP) waves. The proposed MIMO design has a whole ground plane without defective structures, which offers the same voltage level in the antenna configuration. Without integrating any decoupling element, the dual CP MIMO antenna offers good isolation among elliptical-shaped elements.

## 2. Antenna Configuration

**Figure 1:**
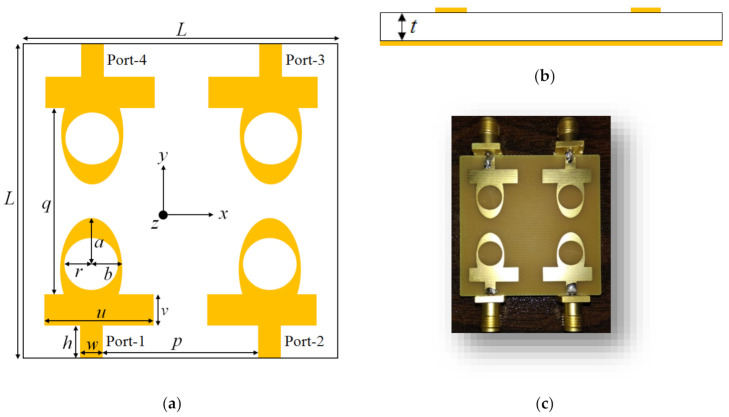
Layout of the proposed dual CP MIMO antenna: (**a**) top view; (**b**) side view; and (**c**) fabricated prototype.

Figure 1a presents a layout of the proposed CP MIMO antenna. The antenna is designed on the FR-4 dielectric substrate with a thickness (*t*) of 1.6 mm, relative permittivity (*ε_r_*) of 4.4, and loss tangent (tan *δ*) of 0.02. The MIMO antenna consists of four elliptical-shaped elements printed on the top side of the dielectric substrate. The four radiators are configured as a mirrored-image pattern, where the element 2 is a mirror image of element 1, and elements 3 and 4 are the mirror images of elements 1 and 2. The resonating elements are excited using 50-Ω microstrip feed lines. The S_11_ and axial ratio characteristics of all four elements are identical due to their same geometry and size. The side view of the MIMO antenna is displayed in Figure 1b. The proposed MIMO antenna was developed using the 3D EM tool (ANSYS HFSS^®^), and the dimensions of the antenna are shown in Table 1. The overall size of the CP MIMO antenna is 40 mm × 40 mm × 1.6 mm.

### 2.1. Development Process

**Figure 2:**
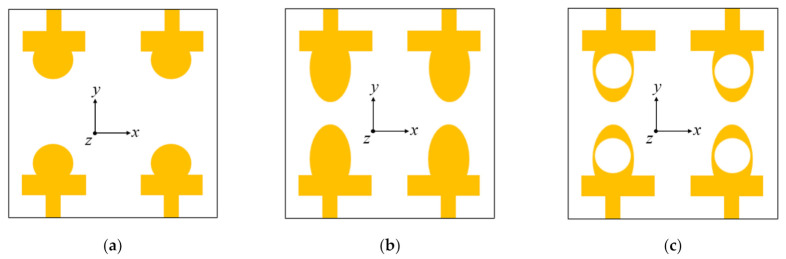
Design stages of the proposed CP MIMO antenna: (**a**) stage 1; (**b**) stage 2; and (**c**) stage 3.

The development procedure of the presented CP MIMO antenna is given in Figure 2. The MIMO antenna comprises four identical elliptical-shaped radiators arranged in a mirror-image pattern. Hence, the design method of all the resonating elements will be the same. In order to have a clear understanding of the radiator design, the design stages of element 1 are explained in this section.

**Figure 3:**
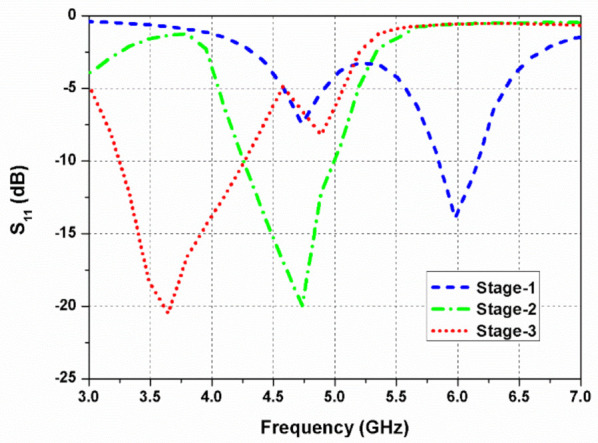
Simulated reflection coefficients of the evolution steps.

**Figure 4:**
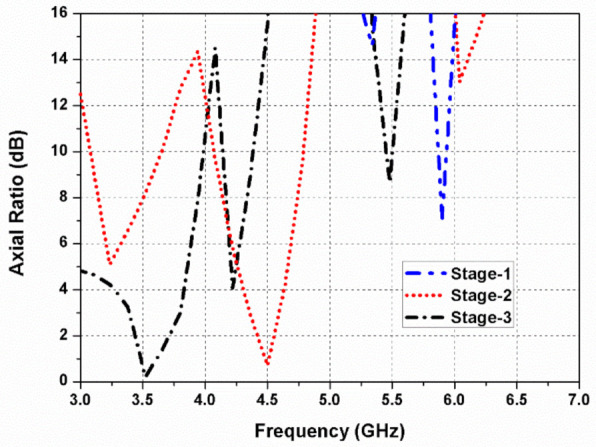
Simulated axial ratio of the evolution steps.

As shown in Figure 2a, the antenna element (Ant. 1) consists of a 50-Ω feed line (*h* × *w*) and a circular radiator. Ant. 1 resonates at a frequency of 6 GHz, illustrated in Figure 3. However, the objective is to cover the 5G (sub-6 GHz) band, so the resonating band must be shifted to the lower frequency side. In the second step, as shown in Figure 2b, the circular radiator is transformed into an elliptical resonator (Ant. 2) to increase the surface current path of the antenna, which shifts resonating frequency towards the lower side. Additionally, the rectangular stub is lengthened towards the right side (along the +*x*-axis direction in the antenna element 1), which balances the magnitude and 90° phase variance among the horizontal (*E_x_*) and vertical (*E_y_*) fields (see Figure 4). It also improves impedance matching between the elliptical radiator and the feed line.

Further, in Figure 2c, a circular slot is etched out from the elliptical radiator (Ant. 3) to shift frequency to the (3.4–3.8 GHz) sub-6 GHz band. The path of the surface current is lengthened when a circular slot is loaded in the elliptical patch, shifting the resonant frequency to a lower value. In the elliptical-shaped antenna element, the ratio of (*E_x_*/*E_y_*) is closer to 0 dB with a 90° phase variation among them. The simulated reflection coefficient and axial ratio curves of the design stages (at port 1) are presented in Figure 3 and Figure 4, respectively.

### 2.2. Mutual-Coupling Reduction

The integration of numerous antenna elements into a compact-size wireless system is a challenging task due to high mutual coupling between resonating elements, especially when they share the same ground plane and are placed less than half a wavelength apart [24,25,26,27,28]. The mutual coupling between different elements is mainly affected by the surface current distribution. In MIMO/diversity antennas, the mutual coupling amongst closely packed resonating elements can be decreased by using the decoupling structures. The decoupling structures restrict the flow of the surface current from the exciting port to the other ports. However, the introduction of decoupling techniques such as EBG, parasitic patch, or ground plane defects increases the complexity of the design and profile of the MIMO antenna. A defect in the ground plane can cause undesired radiations on the back side of the antenna and complicate the antenna mounting on the printed circuit board (PCB) [29].

Additionally, the inter-element coupling level can be reduced by increasing the gap between the radiators, which increases the overall antenna size. Compact-sized antennas are preferable for their ease of integration into portable wireless devices, while at the same time, it is necessary to keep mutual coupling to the lowest level for better functioning of the antenna. In the proposed CP MIMO antenna, an optimized gap spacing between the elliptical-shaped elements is obtained to preserve the ARBW of the antenna elements. Altering the gaps (the horizontal antenna spacing (*p*) and the vertical antenna spacing (*q*)) between the elliptical-shaped elements have less of an impact on the impedance bandwidth of the proposed MIMO antenna, but it considerably affects the ARBW.

**Figure 5:**
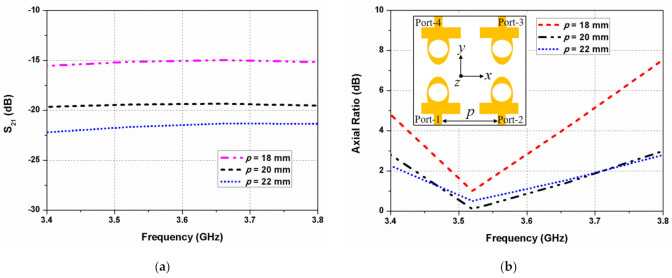
Effect of the horizontal antenna spacing (*p*) on (**a**) S_21_ and (**b**) axial ratio.

**Figure 6:**
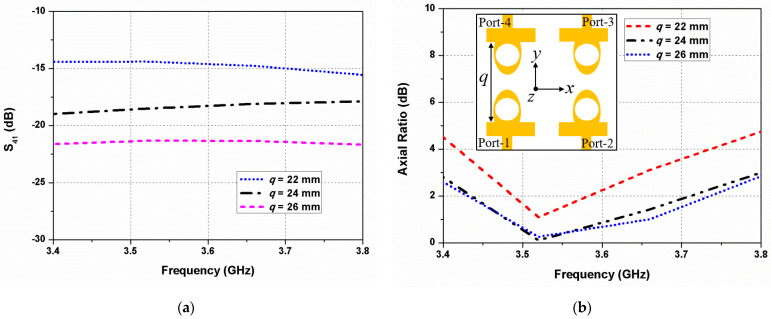
Effect of the vertical antenna spacing (*q*) on (**a**) S_21_ and (**b**) axial ratio.

The horizontal antenna spacing (*p*) between the radiating elements 1 and 2 is varied, and its effect on the mutual coupling and 3-dB ARBW is illustrated in Figure 5. The isolation improves significantly when the spacing between the elements increases, while a small gap increases the mutual coupling, as shown in Figure 5a. In Figure 5b, it can be seen that when *p* = 18 mm, the 3-dB ARBW of the antenna decreases to 3.46–3.61 GHz due to increased inter-element coupling. The mutual coupling and axial ratio deviations with the vertical antenna gap (*q*) between the elements 1 and 4 are shown in Figure 6. It is evident that *q* affects significantly the mutual coupling and 3-dB ARBW of the designed antenna. The mutual coupling increases with less spacing among the resonators. A large gap, however, is not preferred since it increases the overall size of the antenna.

### 2.3. Circular Polarization Mechanism

**Figure 7:**
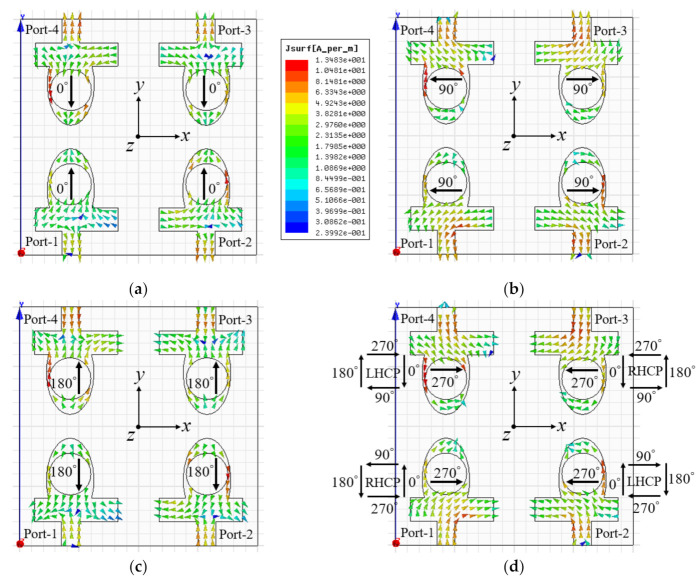
Surface current distribution at 3.6 GHz (**a**) *ωt* = 0°; (**b**) *ωt* = 90°; (**c**) *ωt* = 180°; and (**d**) *ωt* = 270°.

Figure 7 illustrates the simulated current distributions of the proposed dual CP antenna (at 3.6 GHz) when all resonators are excited at the same time. The current distributions are shown at four different time phases (*ωt* = 0°, 90°, 180°, and 270°). At *ωt* = 0°, the dominant current vectors (at resonator 1 and resonator 2) flow in the +*y*-axis, whereas at *ωt* = 180°, the dominant current vectors are in −*y*-direction.

At *ωt* = 90° and 270°, the current vectors at resonator 1 and resonator 2 are equivalent in magnitude and opposite in phase. The current vectors (at port 1) flow in the −*x*-direction at *ωt* = 90°, whereas at *ωt* = 270°, the predominant current vectors flow in +*x*-direction. Alternatively, at *ωt* = 90°, the predominant current vectors (at port 2) flow in the +*x*-direction, whereas at *ωt* = 270°, the predominant current vectors flow in −*x*-direction. The current at resonator 1 and resonator 3 rotates in the anticlockwise direction, which illustrates RHCP functioning of the resonators 1 and 3, as illustrated in Figure 7d. The current vectors at resonator 2 and resonator 4 rotate in the clockwise direction, which demonstrates LHCP functioning of the resonators 2 and 4. It is noted that the directions of the current vectors on the neighbouring elliptical resonators are opposite, and therefore they cancel each other, suppressing inter-element coupling at the resonating frequency.

## 3. Results and Discussion

**Figure 8:**
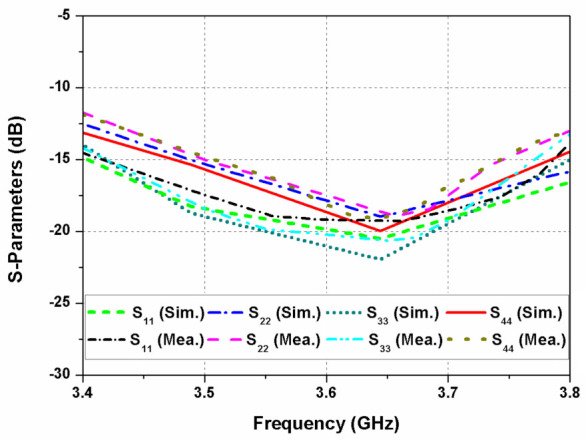
Reflection coefficients of the designed MIMO antenna.

**Figure 9:**
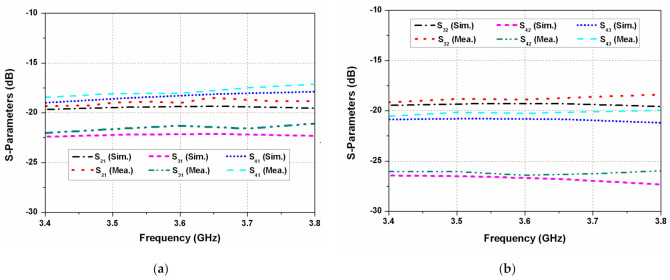
S-parameters of the designed antenna: (**a**) when port 1 is excited; (**b**) when port 2/port 3 is excited.

The proposed dual CP MIMO antenna is fabricated and measured to verify its performance. Figure 1c shows the photograph of the prototype antenna. The S-parameters of the proposed dual CP MIMO antenna are measured using the Anritsu MS4644B vector network analyzer. The reflection coefficients of the antenna elements 1, 2, 3, and 4 are shown in Figure 8. It can be observed that the reflection coefficients are below −10 dB for 3.4 to 3.8 GHz range. The simulated and measured mutual coupling of the antenna are shown in Figure 9a,b. The measured isolation is higher than 18 dB for the whole frequency range. A reasonable agreement is observed between the simulated and experimental results of the MIMO antenna.

**Figure 10:**
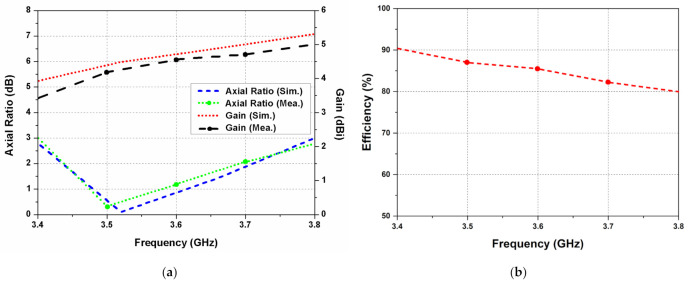
(**a**) Axial ratio and gain of the dual CP MIMO antenna; (**b**) radiation efficiency of the proposed antenna.

Figure 10a depicts the simulated and measured axial ratio and gain values for the designed CP MIMO antenna. An axial ratio of less than 3 dB is observed between 3.4 to 3.8 GHz band. A maximum gain of 5 dBi is obtained at a frequency of 3.8 GHz. While performing measurements of one antenna element, the other antenna elements are matched with 50-Ω loads. Figure 10b depicts the measured radiation efficiency of the designed CP MIMO antenna. A maximum efficiency of 90% is obtained at a frequency of 3.4 GHz. A small discrepancy in the experimental and simulated results is due to the soldering of SMA connectors and measurement errors.

**Figure 11:**
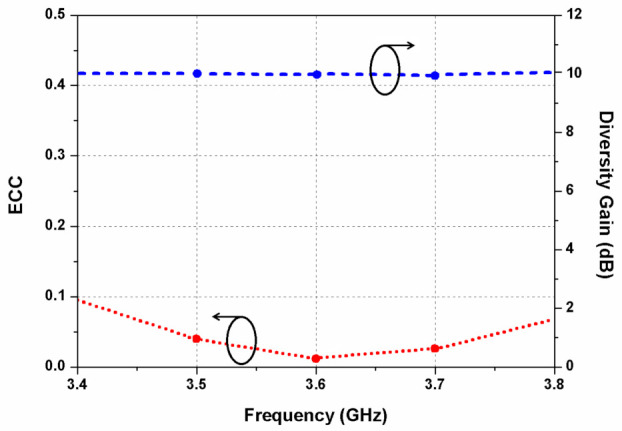
Measured ECC and DG of the dual CP MIMO antenna.

Figure 11 presents the envelope correlation coefficient (ECC) and diversity gain (DG) of the proposed dual CP MIMO antenna. The ECC can be expressed as [30]

(1) $$\rho_{e}=\frac{{\left|\int \int \left[\vec{F_{1}}\left(\theta ,\;\varphi \right)\vec{F_{2}}\left(\theta ,\;\varphi \right)\right]\;d\Omega \right|}^{2}}{\int \int {\left|\vec{F_{1}}\left(\theta ,\;\varphi \right)\right|}^{2}d\Omega \int \int {\left|\vec{F_{2}}\left(\theta ,\;\varphi \right)\right|}^{2}d\Omega }$$

where $\vec{F_{i}}\left(\theta ,\;\varphi \right)$ is the three-dimensional radiation pattern of the antenna element when the *i*th port is excited. The ECC value remains below 0.1 for the entire frequency band, which indicates a very low correlation between the antenna elements or good MIMO diversity performance.

The DG of the MIMO design can be evaluated using [18]

(2) $$\mathrm{DG}=10\sqrt{1-{\left(\rho_{e}\right)}^{2}}$$

Due to the different polarization of adjacent elliptical resonators, the DG of the proposed antenna is about 10 dB within the operating band.

**Figure 12:**
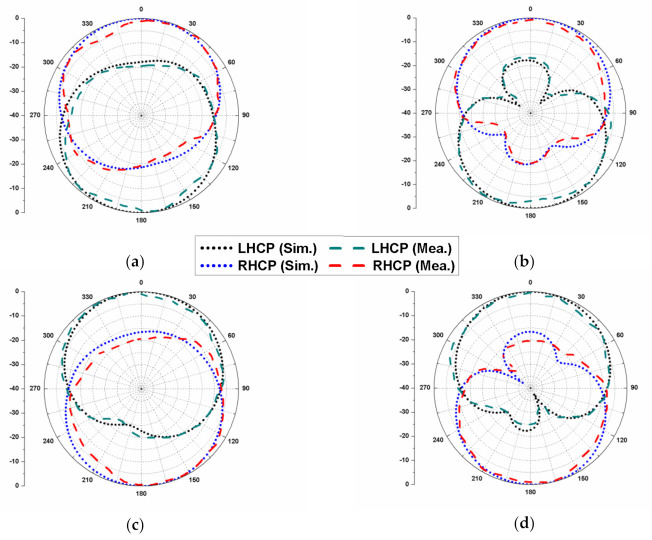
Radiation patterns of the dual CP MIMO antenna at 3.6 GHz: (**a**) port 1, *φ* = 0°; (**b**) port 1, *φ* = 90°; (**c**) port 2, *φ* = 0°; and (**d**) port 2, *φ* = 90°.

The simulated and measured radiation patterns (at 3.6 GHz) of the proposed dual CP MIMO antenna are given in Figure 12. The antenna element 1 shows RHCP behavior when port 1 is excited as shown in Figure 12a,b, whereas the antenna element 2 shows LHCP characteristics when port 2 is excited as shown in Figure 12c,d. In the same way, the antenna element 2 emits RHCP waves, while element 4 emits LHCP waves, which validates the dual CP behavior of the MIMO antenna. The no-defect ground plane and opposite polarization of the antenna elements reduce the correlation between the resonating patches and make it possible to locate them closely to obtain a compact geometry.

## 4. Performance Comparison

Table 2 shows the performance comparison of the proposed dual CP MIMO antenna and the reported CP MIMO/diversity antennas. The MIMO antennas reported in [14,15] showed single-sense circular polarization behavior, poor inter-element coupling, and low gain. The dual CP MIMO antenna designs presented in [16,18] were two-element designs with relatively large size. The two-element MIMO antennas presented in [17,19] showed better isolation, but the ground planes of their resonating elements were not linked to each other. The antenna in [20] consisted of four elements, but it had a larger size and an I-shaped decoupling structure for isolation. The MIMO antenna proposed in [21] consisted of four resonators, but it had a larger size, small ARBW, and complicated geometry. Additionally, the ground surfaces of the antenna elements [14,15,16,17,18,19,20,21] had some defect or truncations to obtain circular polarization. In the reported CP MIMO antenna designs, modified ground planes and complex decoupling methods were used to improve isolation. The comparison shows that the proposed dual CP MIMO antenna is compact in size with full bandwidth coverage with the axial ratio and impedance performances. Additionally, the proposed CP MIMO antenna consists of four resonators and a whole ground plane without any defects. Additionally, no extra decoupling element between the radiators is used to improve isolation. Since the antenna elements 1 and 2 are mirror images of each other, and since the antenna elements 3 and 4 are mirror images of the antenna elements 1 and 2, the directions of the coupling current vectors are opposite to each other. Due to the mirror-image arrangement of the elliptical-shaped elements, which supports polarization diversity, the proposed MIMO antenna could achieve high isolation with very small element separation distance. Hence, the MIMO antenna can be easily integrated with planar microwave circuits owing to its simple geometry, low-profile, and a common no-defect ground plane.

## 5. Conclusions

A planar dual CP MIMO/diversity antenna is presented for the sub-6 GHz frequency band. The antenna is composed of four identical elliptical-shaped resonators, which are configured as a mirror-image design to introduce polarization diversity. The antenna features axial ratio and impedance bandwidths of 3.4–3.8 GHz, ECC < 0.1, DG ~10 dB, isolation > 18 dB, and a peak gain of 5 dBi. The proposed CP MIMO antenna supports both types of circular polarization, where elements 1 and 3 offer RHCP and the elements 2 and 4 offer LHCP. The MIMO antenna can be simply integrated with portable, monolithic, microwave-integrated circuits owing to its compact geometry and its simple ground plane without any defects. The proposed dual CP MIMO antenna can be employed for sub-6 GHz 5G applications.

## Figures and Tables

**Table 1:** Design particulars of the dual CP MIMO antenna.

Dimensions	Value (mm)	Dimensions	Value (mm)
*L*	40	*a*	7
*t*	1.6	*b*	4
*h*	4	*r*	3.5
*w*	3	*p*	20
*u*	14	*q*	24
*v*	4		

**Table 2:** Performance comparison with the reported CP MIMO antennas.

Ref.	Design Methodology	No. of Resonators	Polarization	(S_11_ ≤ −10 dB) Band (GHz)	3-dB Axial Ratio Band (GHz)	Antenna Size (mm^3^)	Connected Grounds	Isolation (dB)	Edge-to-Edge Distance b/w the Radiators (mm)	ECC	Peak Gain (dB)	Design Complexity
[14]	Front-to-back radiators	2	LP/ RHCP	5.4–6.1	5.8, 5.9, 6	25 × 30 × 1.524	No	>13	1.524	<0.06	4.3	High
[15]	Non-identical resonators	3	LP/ RHCP	5.4–6.2	5.61–5.7	29 × 48 × 1.6	Yes	>15	8.35	<0.01	4	High
[16]	Wide slot	2	LHCP/ RHCP	2–4.76	2–3.7	60 × 60 × 1.6	Yes	>15	15.2	---	4	Moderate
[17]	Shared radiator	2	LHCP/ RHCP	1.4–8.73	3.74–8.8	32 × 32 × 1	No	>20	32	<0.5	3.8	High
[18]	Stubs and defected ground	2	LHCP/ RHCP	2.47–2.55	2.5–2.66	100 × 150 × 0.8	Yes	>20	~61	<0.003	6.1	Moderate
[19]	Mirrored-image	2	LHCP/ RHCP	5.2–6.3	5.2–6.3	13.7 × 36.2 × 0.813	No	>22	23	<0.002	5.8	Moderate
[20]	Mirrored-image	4	LHCP/ RHCP	4–13	4.2–8.5	70 × 68 × 1.6	Yes	>18	36	<0.25	6.4	Moderate
[21]	Mirrored-image	4	LHCP/ RHCP	3.4–3.8	3.46–3.7	60 × 60 × 1.6	Yes	>19	43	<0.12	4.5	High
Prop.	Mirrored-image	4	LHCP/ RHCP	3.4–3.8	3.4–3.8	40 × 40 × 1.6	Yes	>18	20	<0.1	5	Low

## Data Availability

Not applicable.
